# A Microfluidic Hanging-Drop-Based Islet Perifusion System for Studying Glucose-Stimulated Insulin Secretion From Multiple Individual Pancreatic Islets

**DOI:** 10.3389/fbioe.2021.674431

**Published:** 2021-05-12

**Authors:** Patricia Wu Jin, Nassim Rousset, Andreas Hierlemann, Patrick M. Misun

**Affiliations:** Bio Engineering Laboratory, Department of Biosystems Science and Engineering, ETH Zürich, Basel, Switzerland

**Keywords:** glucose-stimulated insulin secretion (GSIS), pancreatic islets, microfluidics, hanging drops, perifusion systems, organ on chip

## Abstract

Islet perifusion systems can be used to monitor the highly dynamic insulin release of pancreatic islets in glucose-stimulated insulin secretion (GSIS) assays. Here, we present a new generation of the microfluidic hanging-drop-based islet perifusion platform that was developed to study the alterations in insulin secretion dynamics from single pancreatic islet microtissues at high temporal resolution. The platform was completely redesigned to increase experimental throughput and to reduce operational complexity. The experimental throughput was increased fourfold by implementing a network of interconnected hanging drops, which allows for performing GSIS assays with four individual islet microtissues in parallel with a sampling interval of 30 s. We introduced a self-regulating drop-height mechanism that enables continuous flow and maintains a constant liquid volume in the chip, which enables simple and robust operation. Upon glucose stimulation, reproducible biphasic insulin release was simultaneously observed from all islets in the system. The measured insulin concentrations showed low sample-to-sample variation as a consequence of precise liquid handling with stable drop volumes, equal flow rates in the channels, and accurately controlled sampling volumes in all four drops. The presented device will be a valuable tool in islet and diabetes research for studying dynamic insulin secretion from individual pancreatic islets.

## Introduction

Langerhans islets are micro-organs in the pancreas, which secrete hormones that help maintain glucose homeostasis in the human body. The islets are composed of different endocrine cell types, of which pancreatic beta-cells are the most common ones. They release insulin in a highly dynamic, bi-phasic and pulsatile manner in response to elevated glucose levels in the blood (In’t Veld and Marichal, 2010). Studying this highly dynamic process of glucose-stimulated insulin secretion (GSIS) of pancreatic islets can give insights into the insulin release mechanisms of healthy and diabetic islets. The study of the islet functionality is important for the assessment of anti-diabetic medication in drug-screening applications and for the determination of islet quality for islet transplantation (Papas et al., 2009). However, reliable technological methods and platforms to characterize islets as well as relevant and reproducible islet model systems are scarce.

Reaggregated human islet microtissues, derived from native human islets, have shown to be robust and reproducible biological model systems for drug screening due to their functionality, uniform size and cellular composition (Misun et al., 2020). Furthermore, microtechnology can produce small-volume microfluidic devices, which allow for studying GSIS dynamics of individual islets. Unlike conventional well plate formats, microfluidic platforms allow for a precise control of small liquid volumes and of the microenvironment around cells and tissues, which helps to better mimic physiologically relevant conditions (Dittrich and Manz, 2006; El-Ali et al., 2006; Bhatia and Ingber, 2014; Convery and Gadegaard, 2019).

Recently, microfluidic perifusion systems were developed to culture and assess pancreatic islet functionality *in vitro* (Wang et al., 2010; Li et al., 2013; Castiello et al., 2016; Lee et al., 2018). This assessment was done by measuring the response of islets upon dosage of physiological glucose concentrations using various imaging or biochemical assays (Rocheleau et al., 2004; Cabrera et al., 2008; Mohammed et al., 2009; Adewola et al., 2010; Godwin et al., 2011; Shackman et al., 2012; Heileman et al., 2015; Brooks et al., 2016; Nourmohammadzadeh et al., 2016; Lenguito et al., 2017). Also, commercially available islet perifusion systems have been used in different GSIS studies (Buchwald et al., 2018; Glieberman et al., 2019). However, the presented devices and measurements are either limited in temporal resolution or require pooling of multiple islets to achieve quantifiable insulin concentrations.

Dedicated devices are needed to resolve the highly dynamic and oscillatory secretion pattern of islets (Ritzel et al., 2003; Nunemaker et al., 2009; Yi et al., 2015). Sufficient resolution could be achieved with more complex experimental setups by making use of electroosmotic flow and an on-line electrophoresis immunoassay, which allowed for resolving the insulin secretion pattern of single islets (Roper et al., 2003; Shackman et al., 2005; Dishinger and Kennedy, 2007; Dishinger et al., 2009), or by performing an on-chip immunoassay using fluorescence anisotropy (Glieberman et al., 2019; Adablah et al., 2020). Despite these technological advances, reports on applications of microfluidic perifusion systems for studying islets secretion are still few. One of the reasons is that microfluidic devices for islet research are mostly very complex and are, therefore, not easy to operate, which limits their use by non-experts. Moreover, simple microfluidic perifusion systems mostly lack the design components to achieve quantification of insulin secretion at high temporal resolution. High liquid sampling rates alone do not automatically lead to high-resolution measurements. Flow conditions and an optimal flow profile also need to be considered to minimize dilution, dispersion and diffusion effects of the secreted hormones in a comparably large liquid volume.

Microfluidic perifusion systems for the study of the release characteristics of single pancreatic islets need to meet certain requirements. (i) The system should have a simple microfluidic setup that allows for stable immobilization of single, isolated islets in small and perfused culturing compartments; (ii) it needs to feature precise fluid control and optimal flow profiles to ensure short delays and minimal analyte dispersion, so that rapid changes in insulin secretion patterns can be resolved; (iii) there should be minimal dilution the secreted analyte to reach the limit of detection (LOD) for standard ELISA quantification; (iv) the system should support a high sampling frequency in combination with a simple analysis method for small-volume liquid samples; (v) the system should allow for parallelization of measurements in order to increase the experimental throughput.

Here, we describe a new platform featuring significant advances over the recently presented microfluidic hanging-drop-based islet perifusion platform that was used to study alterations in the dynamics of insulin secretion of single pancreatic islet microtissues at high temporal resolution (Misun et al., 2020). Already the previous perifusion platform relied on important advantages of the hanging-drop technology (Frey et al., 2014; de Groot et al., 2016): the completely open microfluidic chip design allowed for easy loading and retrieval of islets for subsequent analysis; islet microtissues were kept at the liquid-air interface at the bottom of a hanging drop; optimal oxygen supply was ensured, which is important to closely mimic the *in vivo* environment of islets (Ohta et al., 1990; Dionne et al., 1991; Buchwald, 2011; Lo et al., 2012; Nourmohammadzadeh et al., 2013); the relatively small surface area of the chip reduced analyte ad- and absorption (Toepke and Beebe, 2006); high sampling rates in combination with the dedicated chip design enabled to resolve the physiologically characteristic biphasic pattern and pulsatile insulin release of human islet microtissues.

The new generation of hanging-drop-based islet perifusion platforms was designed to further reduce operational complexity and to increase experimental throughput. The new system features a microfluidic network of four parallel hanging drops – each hosting an individual pancreatic islet, and a needle-type control drop that automatically adjusts and equilibrates the size of all hanging drops in the microfluidic network (Birchler et al., 2016; Misun et al., 2018). The system was designed to maintain slip-boundary conditions at the air-liquid interface for rapid liquid turnover and to enable aliasing-free sampling of the release dynamics of all four individual islets.

## Materials and Methods

### Reaggregated Primary Human Islets

3D InSight Human Islet Microtissues were obtained from InSphero AG (Schlieren, Switzerland) and maintained in 3D InSight Human Islet Maintenance Medium, also from InSphero AG (Schlieren, Switzerland) at 37°C and 5% CO_2_.

### Fabrication of the Microfluidic Hanging-Drop Chip

Microfluidic chips were fabricated using a 3D printed mold (Protolabs, Feldkirchen, Germany) and casting PDMS (Sylgard 184, Dow Corning GmbH, Wiesbaden, Germany). The 3D mold was designed in Autodesk Inventor. The smallest features in the xy-plane were 200 μm, and 100 μm in the z-plane. The mold was printed with the Accura SL5530 high resolution plastic material with a natural surface finish. PDMS was prepared by mixing the elastomer and curing agent in a 10:1 ratio. PDMS was poured onto the 3D mold resulting in a 3 mm thick layer and cured overnight at 50°C. Individual chips were cut, and access holes for the fluidic connections were punched with biopsy punches with a diameter of 0.41 mm for the inlet and outlet and 1.40 mm diameter for the hole at the control drop side.

The PDMS chip was bonded by plasma activation of the bonding surfaces to a microscope slide featuring access holes of 1.2 mm for inlet and outlet, and 2.0 mm for the needle-type valve. Lastly, a NanoPort assembly (N-333, Idex Health & Science, Wertheim, Germany) was glued onto the glass slide and centered above the control drop. A TE 33 GA chamfer gray metal connector (Nordson Schweiz AG, Vilters, Switzerland) was used as a needle-type valve, which was inserted in a F-242 ferrule and affixed to the NanoPort head fitting.

### Chip Preparation and Liquid Loading

The microfluidic chip was cleaned with soap, water, and ethanol, and dried with pressurized air. The needle-type valve was inserted in the NanoPort so that the ferrule sealed the PDMS hole, and the perifusion tubes were connected to the inlet and outlets. The hydrophobic PDMS chip was loaded with prewarmed (37°C) 3D InSight Krebs Ringer HEPES Buffer (KRHB), 0.5% BSA (InSphero AG, Schlieren, Switzerland) by pipetting KRHB into the drop structures, and the liquid was distributed to the microfluidic channel structures with the help of a thin metal needle. The filled hanging-drop chip was then affixed on a customized chip holder placed in a Nunc OmniTrayBox (Thermo Fisher Scientific, Reinach, Switzerland).

### Microtissue Loading

Single microtissues were aspirated from the GravityTRAP plates (InSphero AG, Schlieren, Switzerland) with a 10 μL pipette and loaded into each of the drop compartments in a standing-drop configuration via contact transfer. The chip was then flipped to a hanging-drop configuration, and the drop height was defined by the length of the needle-type valve, which was protruding approx. 600 μm from the rim structure, so that well-defined drops of about 600 μm height were formed and maintained during perifusion.

### Experimental Setup and Perifusion System

Supplementary Figure 1A shows the experimental setup. The chip was placed inside an incubator (Teco 20, Selutec GmbH, Hechingen, Germany) set at 37°C. A liquid reservoir on the chip holder and a wet cotton pad in the OmniTrayBox increased the humidity to minimize evaporation. Additionally, the OmniTrayBox was sealed with a lid and parafilm to further reduce evaporation and prevent air flow around the chip and the hanging drops.

Two 10 mL glass syringes (ILS, Ilmenau, Germany) were mounted to syringe pumps (neMESYS, Cetoni GmbH, Korbussen, Germany). Syringes were connected via standard Luer-lock syringe-tubing connectors (TE 27 GA 90° bent, APM Technica AG, Heerbrugg, Switzerland), polytetrafluoroethylene (PTFE) tubing (ID 0.3 mm, OD 0.6 mm, Bola GmbH, Grünsfeld, Germany) and metal connecting pieces, obtained from standard Luer-lock syringe-tubing connectors (TE 27 GA 90° bent, APM Technica AG, Heerbrugg, Switzerland), to a vacuum-connected splitter (Supplementary Figure 1C) that was used to connect multiple syringes to the common inlet of the chip.

A peristaltic pump (peRISYS-S, Cetoni GmbH, Korbussen, Germany) was used to withdraw liquid from the chip. Upstream, four peristaltic tubings (Tygon S3 E-LFL, ID 0.27 mm, wall 0.91 mm, Idex Health & Science GmbH, Wertheim, Germany) were connected as described above to the four outlets of the chip. Downstream, the tubings were connected to isolated metal tubing connectors / sampling needles (TE 27 GA straight, APM Technica AG, Heerbrugg, Switzerland) that were affixed to a customized sampling needle holder at the sampling arm of the rotAXYS (Cetoni GmbH, Korbussen, Germany).

An additional peristaltic tube with a larger diameter (Tygon LMT-55, ID 0.51 mm, wall 0.91 mm, Idex Health & Science GmbH, Wertheim, Germany) was used to withdraw liquid from the control drop. The larger inner diameter enabled the quick removal of the extra volume from the system. This peristaltic tube was connected to the needle-type valve by a metal connecting piece (TE 25 GA bent, APM Technica AG, Heerbrugg, Switzerland) and a short flexible silicone tubing (Tygon LMT-55, ID 0.38 mm, wall 0.91 mm, Idex Health & Science GmbH, Wertheim, Germany).

The inflow rate of the syringe pumps was set to 80 μL min^–1^, and the outflow rate at the peristaltic pump was set to 15 μL min^–1^ for each islet drop. The outflow rate from the control drop was approximately 45-60 μL min^–1^. At this rate, considering the length of the tubing, there was approximately a 5.5 min delay between the switching of different buffers to stimulate the islet microtissues and the collection of the samples. This delay was considered in the perifusion and sampling scripts by adjusting the timing accordingly.

### Automated Liquid Sampling

A programmable positioning and sampling system (rotAXYS, Cetoni GmbH) was used to collect outflow samples at 30 s intervals (7.5 μl samples). The Qmix Elements Software (Cetoni GmbH, Korbussen, Germany) was used to create the perifusion and sampling script. Multiwell plates (384-well, flat bottom, small volume, white, polystyrene, REF 784075, Greiner Bio-one) were prefilled with 3 μL of mineral oil (Ultra for molecular biology, REF 69794, Sigma-Aldrich) to reduce sample evaporation during sampling. The well plate was placed in a customized stage, made of plexiglass, and was used for collecting the samples (Supplementary Figure 1B). With the defined flow rate of 15 μL min^–1^, 7.5 μL of sample volume were collected.

### Perifusion GSIS

Two 10 mL glass syringes were loaded with KRHB + 0.5% BSA with 2.8 × 10^–3^ M glucose and 16.7 × 10^–3^ M glucose. Prior to loading into the syringes, the medium was equilibrated and degassed at 37°C overnight. The buffer solutions were consecutively perfused through the hanging-drop chip in the following order; 30 min 2.8 × 10^–3^ M glucose for equilibration, 5 min 2.8 × 10^–3^ M glucose for baseline secretion, 29 min 16.7 × 10^–3^ M glucose for stimulation secretion, and 32 min 2.8 × 10^–3^ M glucose for post-GSIS secretion.

### Quantification of Insulin

The secreted insulin was quantified using the Insulin Ultra-sensitive Assay Kit (CISBIO). The ELISA assay was miniaturized from 10 μL to 5 μL (Supplementary Figure 2A). The ELISA reagent was directly added to the samples at a ratio of 1:1. The sampling plate was incubated overnight at room temperature, and the readout was performed using a microplate reader (Infinite M1000, TECAN, Männedorf, Switzerland).

### Microfluidic Characterizations

The drop height of an islet compartment was monitored over 73 min of perifusion at 37°C. An SU-8 ring with hydrophilic coating (thickness of 40 μm, rim width of 50 μm and inner diameter of 400 μm) was loaded into the hanging drop. It was used as a feature that provided contrast and could be detected by a microscope, so that the focal plane could be determined. The SU-8 ring sedimented to the liquid-air interface after loading. The z-position of the ring was monitored with an inverted wide-field microscope (Leica DMI6000, Leica Microsystems, Switzerland) using a 10x objective and a 0.70x c-mount. Bright-field images were taken with a Retiga SRV camera from QImaging (01-RET-SRV-F-M-12-C) approximately every 3 s. A software-based autofocusing was executed before every image acquisition, and the focal planes of the images were recorded over time. The frequency of the hanging-drop size fluctuations and the frequency at which air-liquid fractions were taken from the needle-type valve were tracked using a camera (Sony α3000 with E PZ 16-50mm f/3.5/5.6 OSS E-mount lens) and a Dino-Lite USB microscope camera with the DinoCapture 2.0 software (Dunwell Tech., Inc., United States). The videos were analyzed using ImageJ to obtain the mean intensity profile in the ROI, and a Fast Fourier Transform (FFT) analysis was performed using the Origin software (OriginLab, Northampton, MA, United States).

The fluidics were further characterized by injecting 5 μM of fluorescein sodium salt (NaFl, BioReagent, REF 46960, Sigma-Aldrich) with small needles (TE 30 GA 90° bent, APM Technica AG, Heerbrugg, Switzerland), which were positioned close to the liquid-air interface of the drops, directly into the hanging drops. KRHB was perfused with an inflow rate of 80 μL min^–1^, and the outflow rate was set to 15 μL min^–1^. NaFl was injected at 500 nL min^–1^. Liquid samples were collected in a microwell plate (384-well, flat bottom, small volume, white, polystyrene, REF 784075, Greiner Bio-one) that was prefilled with 3 μL of mineral oil (Ultra for molecular biology, REF 69794, Sigma-Aldrich) and analyzed using a microplate reader (Infinite M1000, TECAN, Männedorf, Switzerland).

### Data Analysis

For each given sampling interval, the average insulin secretion rate per microtissue (fmol IEQ^–1^ min^–1^) was calculated from the known flow rate and the insulin concentration in the sample. This value was normalized to the islet microtissue size in IEQs, with one IEQ corresponding to the volume of a sphere with a diameter of 150 μm.

### Diffusion-Convection Transport Model

Diffusive-convective transport of molecules through the liquid phase and PDMS was modeled numerically using a finite-element method. 3D model designs of a single hanging-drop chip were produced in Autodesk Inventor and imported into COMSOL Multiphysics^®^ v. 5.4 software (COMSOL AB, Stockholm, Sweden). The 3D model design included a single hanging drop with connecting channels to the inlet and outlet. The drop diameter was 2 mm, and the drop height varied from 0.2 mm to 1 mm. A spherical model islet of 150 μm diameter was located at the bottom of the hanging drop (see also Misun et al., 2020).

The fluid dynamics model included the viscosity and density of the medium at 37°C, which were assumed to be identical to that of water. Slip and no-slip boundary conditions were assumed at the liquid-air and liquid-PDMS interfaces, respectively. A constant flow and a null pressure were assumed at the inlet and outlet boundaries. These data were used to model the convective transport of insulin.

The diffusion-convection model was applied for insulin with a diffusion coefficient of 1.5 × 10^–10^ m^2^ s^–1^ (Buchwald, 2011). A short insulin secretion burst of an islet was simulated. A total release of 2.5 fmol IEQ^–1^ insulin within one burst was simulated, and a fast secretion within 1 s was assumed, so that the secretion averaged 5 fmol IEQ^–1^ min^–1^ over one sampling interval of 30 s (Misun et al., 2020). The concentration at the outlet was probed over time to investigate sampling aliasing due to variations in drop heights, perifusion rates, and islet microtissue positions. A continuous and constant insulin secretion of an islet was simulated for varying drop heights.

## Results

### Self-Regulating Hanging-Drop Network

Islet perifusion systems need to provide a constant flow of media around the islets. Continuous flow and sampling of the perfused media allows for capturing islet-secreted hormones at defined time points. The intervals between these time points should be short enough to resolve the dynamics of hormone release. Closed microfluidic perifusion systems have a defined volume of liquid in the channels, and pressure can be applied to actuate and control the liquid flow. In contrast, open microfluidic systems, such as microfluidic hanging-drop systems, require active and precise regulation of both, inflow and outflow to actuate liquid flow and to keep the volume in the fluidic system stable. Due to limitations in the operation system and inaccuracies of the pumps, it is impossible to generate exactly equal inflow and outflow. Moreover, hanging drops are susceptible to evaporation, so that the drop volumes may change during perifusion.

**FIGURE 1:**
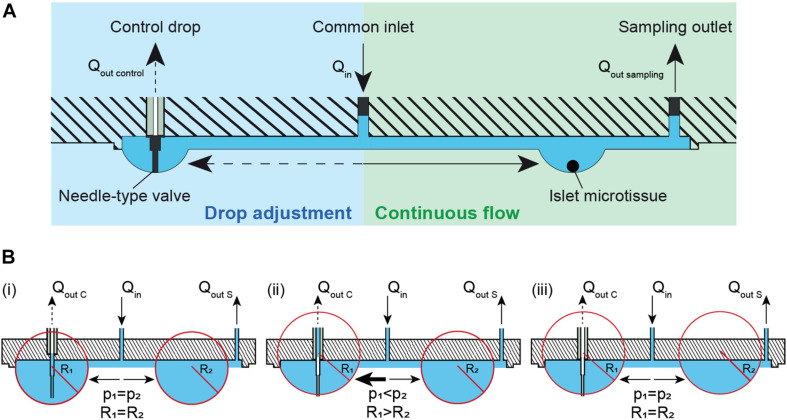
Theory and concept of the automatic hanging-drop-size adjustment in the microfluidic chip. **(A)** The inflow (Q_in_) is split into two flows, one toward the islet drop (right) and the other toward the control drop (left). There is a continuous flow of liquid from the inlet toward the islet drop due to active sampling from the sampling outlet at the rate of Q_out sampling_. An irregular flow is observed from the inlet toward the control drop due to the alternating withdrawal of liquid and air from the needle-type valve at a rate of Q_out control_. **(B)** Self-regulation of drop heights between two interconnected hanging drops. Liquid is constantly added into the system at a rate of Q_in_ and withdrawn through the sampling outlet at a rate Q_out S_ and through a needle-type valve at a rate of Q_out C_. (i) Two hanging drops in equilibrium with identical Laplace radius and pressure. The inflow is evenly distributed to the two drops. As the tip of the needle-type valve is immersed in the drop, liquid removal through the valve will occur. As a consequence, (ii) the size and the Laplace pressure of the two drops becomes different with drop radius and pressure being inversely correlated. Due to the pressure difference, there is an increased flow toward the control drop (left) with the lower pressure. As soon as the tip of the needle-type valve is exposed to air, only air is withdrawn. The control drop remains stable at the height defined by the valve needle length. (iii) In a next step the two drops reach equal Laplace radius and pressure through equilibration through the liquid phase. The system is now stable, and the drop height in control and tissue drop is maintained constant.

Maintaining a constant drop volume in a hanging-drop network under conditions of continuous perifusion is challenging. We developed a method that automatically adjusts the volume and, consequently, the drop size in the fluidic system, which is shown in Figure 1A. Liquid is perfused into the chip through the common inlet at an inflow rate, Q_in_, and liquid is actively withdrawn from the sampling outlet (right) at an outflow rate, Q_out sampling_, where Q_in_> Q_out sampling_. More volume is supplied to the system than is withdrawn in order to compensate for volume losses due to evaporation, and potential inaccuracies of the pumps. The excess volume ensures that the system does not dry out and renders the system stable. Additionally, a needle-type valve in a control drop (left) is used to withdraw any extra volume in the system at a rate of Q_out control_, where Q_out control_ > Q_in_- Q_out sampling_. The control-drop outflow pumping rate, Q_out control_ is set to a high enough rate to pump out excess liquid and/or air depending on the drop radius. The tip of the needle-type valve is set to the desired drop height, so that extra liquid volume is withdrawn from the drop whenever the drop radius extends over the tip of the needle-type valve. This system design provides continuous and uninterrupted flow at a constant flow rate toward the microtissues or islets at the right side, while all the drop sizes in the system are maintained constant through the withdrawal of excess medium from the needle-type outlet in the control drop at the left. Figure 1B shows the principle of how the needle-type valve helps to keep the size of the hanging drops constant through the pressure equilibration across the fluidic network. The pressure equilibration is a consequence of the surface-tension of the curved liquid-air interface (Young-Laplace equation: Δp = 2γ/*R* with *p* denoting pressure, *R* drop curvature radius, and γ surface tension) (Bruus, 2008; Frey et al., 2014; Kundu et al., 2016). In Figure 1B (i), a constant inflow of Q_in_ and two outflows through control drop Q_out C_ and sample drop Q_out S_ are applied. Assuming that the channel resistance is the same toward both, control and sample drops, the inflow is evenly distributed between the two drops that have the same radius and Laplace pressure. In this specific case, the drops extend further than the length of the needle-type valve, so that the valve tip is immersed in liquid. By withdrawing liquid through the needle-type valve, the drop shrinks, until the needle tip gets exposed to air. As the drop shrinks the curvature radius R_1_ increases and the pressure in the drop is reduced (Figure 1B (ii)). The two drops have now different curvature radii and Laplace pressures (R_1_ > R_2_ and p_1_ < p_2_). As a consequence of pressure equilibration across the fluidic network, there is a net flow toward the drop with the lower pressure (p_1_), until both drops are again in equilibrium (Figure 1B (iii)). Both drops remain stable at the height, defined by the valve needle length. The system is now stable, and the drop heights in control and tissue drops are equal and maintained constant.

### Chip Design

**FIGURE 2:**
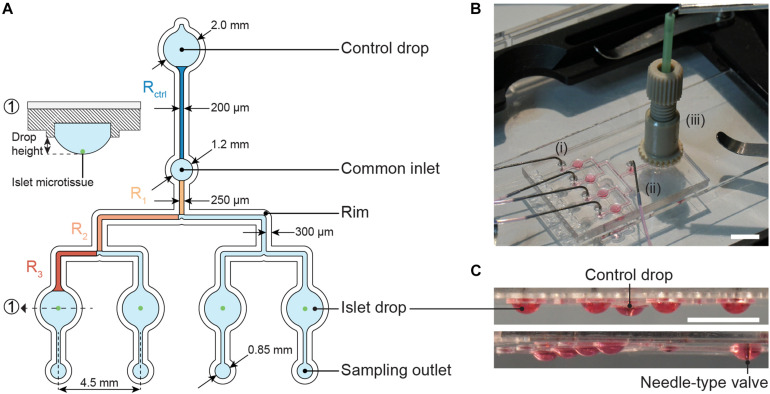
The microfluidic hanging-drop perifusion system. **(A)** Chip layout and dimensions. The fluidic structures (light blue) have a depth of 500 μm, except for the common inlet that has a recess depth of 1 mm. The hydrophobic rim structures (white) define the fluidic channels and hanging drops. They have a height of 250 μm measured from the chip surface. Highlighted channel sections in dark blue and orange were considered for the calculation of the hydraulic resistance in the channels. The channels between common inlet and control drop and between common inlet and islet drops were designed to have the same hydraulic resistance. (1) Cross-sectional view of the islet drop with the islet microtissue at the bottom of the hanging drop. **(B)** Top view of the assembled chip with (i) four parallel outlet tubes, (ii) one common inlet tube, and (iii) a NanoPort assembly with a needle-type valve inserted at the center. Scale bar: 10 mm. **(C)** Side views of the chip with hanging drops visualized with red dye. All hanging drops have equal sizes and shapes. Scale bar: 5 mm.

The microfluidic hanging-drop chip was designed as an open microfluidic system (Frey et al., 2014; Misun et al., 2018, 2020). Figure 2A displays the layout and dimensions of the chip and a cross-sectional view of an islet drop with an islet microtissue. The hanging-drop network is composed of a common inlet, a control drop (2 mm diameter), four sample or islet drops (2 mm diameter), four separate sampling outlets, and the respective interconnecting channels. The fluidic structures are 500 μm deep, and they are defined by a hydrophobic rim structure that serves as a liquid phase guide. Four islet drops are symmetrically connected to the common inlet to ensure equal liquid flow to all drops, and individual sampling outlets allow for simultaneous sampling from all four single islet microtissues in parallel. The common inlet is also connected to a control drop that serves to regulate the size of all hanging drops in the fluidic network. The control drop was designed to have the same geometry as the islet drops to ensure a stable fluidic network with equally shaped hanging drops. The channels connecting the common inlet to the control drop and to the islet drops were designed with the same hydraulic resistances, so that R_control_ = R_drop_, which was important to assure stable operation and equal-size drops during perifusion of the chip. The channel resistances were approximately 5 Pa^∗^s/μL.

The expression for the hydraulic resistance of a rectangular cross section was used to calculate the channel resistance R (Equation 1), where μ is the liquid viscosity, L is the total length of a channel, w is the width, h is the height, while h < w, for details see Figure 2A (Bruus, 2008).

(1) $$\text{R}\approx \frac{12\,\mu \,L}{w\,h^{3}\,\left(1-0.63\,h/w\right)}$$

In the microfluidic channel, the flow rate Q is proportional to the applied pressure: △p = RQ. Flow resistances in fluidic networks can be calculated in analogy to electrical resistances in circuits. The resistance of two channels in series and in parallel can be calculated as R = R_a_ + R_b_ and $\text{R}={\left(\frac{1}{{\text{R}}_{\text{a}}}+\frac{1}{{\text{R}}_{b}}\right)}^{1}$, respectively (Bruus, 2008). Therefore, the total channel resistance from the common inlet to the four sample or islet drops could be obtained according to Equation 2. The channel sections that were used to calculate the channel resistance are highlighted in Figure 2A.

$${\text{R}}_{\text{drop}}={\text{R}}_{1}+{\left(\frac{1}{{\text{R}}_{2}+{\left(\frac{1}{{\text{R}}_{3}}+\frac{1}{{\text{R}}_{3}}\right)}^{-1}}+\frac{1}{{\text{R}}_{2}+{\left(\frac{1}{{\text{R}}_{3}}+\frac{1}{{\text{R}}_{3}}\right)}^{-1}}\right)}^{-1}$$

(2) $$={\text{R}}_{1}+\frac{{\text{R}}_{2}}{2}+\frac{{\text{R}}_{3}}{4}$$

Figure 2B illustrates the assembled chip during operation with tube connections at the common inlet, four sampling outlets, and the NanoPort assembly, which allows for fitting and precise adjustment of the needle-type valve height in the control drop. The needle-type valve height determined the size of all islet drops due to pressure equilibration. Figure 2C shows side views of the chip with evenly sized hanging drops.

### Perifusion Setup and Assay

**FIGURE 3:**
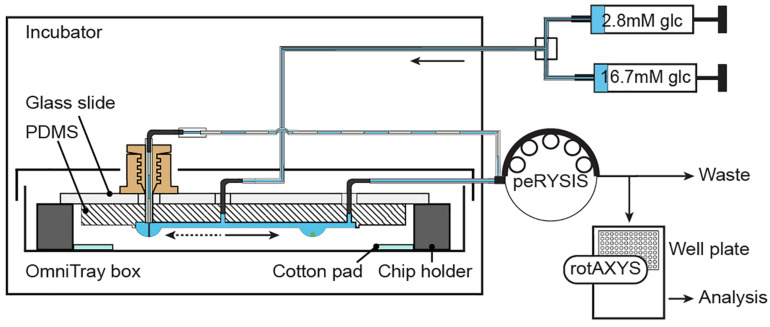
Experimental setup. Two syringes, filled with different media, are connected through a splitter to the common inlet of the chip. Inflow of medium is actuated by using a neMESYS syringe pump. One peRYSIS peristaltic pump was used to actively withdraw liquid from the system at a constant equal flow rate through the 4 sampling outlets and at a higher flow rate (larger tubing diameter) through the control drop needle. Samples were collected into a 384-well plate using the rotAXYS sampling arm (Supplementary Figure 1B) for further downstream analysis. The medium withdrawn from the control drop went to waste.

Figure 3 and Supplementary Figure 1A show the schematic and an image of the islet perifusion setup. The chip was placed inside a 37°C incubator for temperature control. It was placed inside a closed box with parafilm seal, wet cotton pads, and liquid reservoirs in the chip holder in order to reduce evaporation and air flow around the chip. Two syringe pumps, providing media with different glucose concentrations, were connected via a vacuum-connected splitter (Supplementary Figure 1C) to the common chip inlet. The inflow rate was set to 80 μL min^–1^. A peristaltic pump was set to withdraw media at 15 μL min^–1^ for each islet drop, and 45-60 μL min^–1^ for the control drop. A faster withdrawal rate at the control drop was achieved by using a peristaltic tube of bigger diameter, which ensured a quick establishment of the desired drop height. An automated sampling system was used to collect samples via small needles into a 384-well plate (Supplementary Figure 1B). The well plate was prefilled with mineral oil, which helped to prevent evaporation of the samples during the sampling process. The insulin ELISA assay was miniaturized for 7.5 μl samples (Supplementary Figure 2A), and there was no interference of the mineral oil with the insulin ELISA assay (Supplementary Figure 2B). A precise and accurate sampling with the peristaltic pump was achieved (Supplementary Figure 3). 7.5 μl samples were collected at 30 s intervals and used for downstream analysis.

### Fluidic Characterization

**FIGURE 4:**
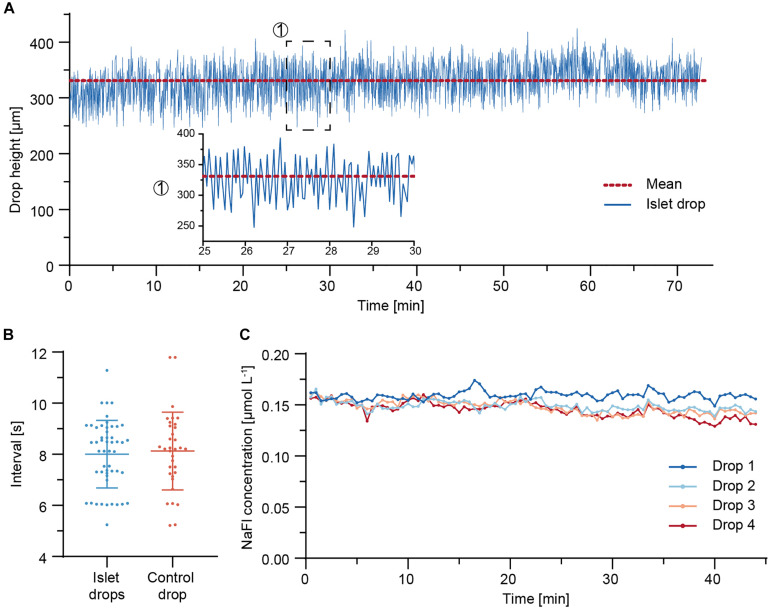
Fluidic system characterization. **(A)** Drop height fluctuations of an islet drop over a 73 min perifusion sequence at 37°C. The red dotted line indicates the mean drop height. A close-up is shown for a 5-min duration. **(B)** Temporal characteristics of drop size fluctuations and of withdrawal of air-liquid fractions through the needle-type valves. Data are mean values ± standard deviation of measurements from eight independent experiments. An average of four to six measurements (5–60 min time window) were taken per experiment. **(C)** Concentration of NaFl, sampled from each hanging drop during a 44 min perifusion sequence. 5 μM NaFl was continuously injected at 500 nL min^– 1^ directly into the hanging drops through a fine needle, while 7.5 μL samples were collected in a well plate at 30 s intervals.

Due to the continuous adjustment of the drop volumes through the needle-type valve, synchronous drop height fluctuations were observed in all four sample drops (Supplementary Movie). The drop height of an islet drop was monitored over 73 min of perifusion. Figure 4A shows the drop height fluctuations. The drop size remained stable over the whole duration of the perifusion, with mean values ± standard deviation of 331 ± 35 μm. Fluctuations in the drop height were expected in the system due to the continuous readjustment of the drop size through liquid removal from the system through the needle-type outlet.

The temporal characteristics of the islet-drop height fluctuations and the temporal characteristics of liquid-air removal from the needle-type valve were compared for different perifusion experiments (Figure 4B). The intervals in observed drop-height fluctuations were highly correlated with those of medium withdrawal through the needle-valve within the same perifusion experiment. For instance, an average interval of 7 s was observed for both fluctuations in the experiment displayed in Figure 4A. The highly correlated temporal characteristics indicate that the fluctuations in drop height were most likely a consequence of medium withdrawal through the needle valve. The temporal characteristics were not affected by the step rotation of the peristaltic pump. However, the fluctuation intervals varied between different experiments. These variations between different perifusion experiments were most likely a consequence of differences in needle-type valve height, which was set manually for every experiment. Shorter fluctuation intervals are expected for using a shorter needle-type valve, as already small volume changes in the system will cause faster and more pronounced changes in drop sizes and heights.

To determine whether the observed drop height fluctuations would affect dilution and dispersion characteristics of analytes within the drops and, consequently, the sampling results of the secreted analytes, 5 μM of fluorescein sodium salt (NaFl) were continuously injected at 500 nL min^–1^ directly into the four sample or islet drops via fine needles, and the corresponding samples were collected in a well plate. Figure 4C shows the concentration of NaFl in the samples collected from each drop. The sampled NaFl concentrations were relatively stable during 44 min of perifusion, with small differences between the drops. The mean values ± standard deviation were 0.159 ± 0.004 μmol L^–1^, 0.149 ± 0.005 μmol L^–1^, 0.148 ± 0.007 μmol L^–1^, and 0.145 ± 0.007 μmol L^–1^ for drops 1–4, respectively. The maximum relative deviation was 5% for drop 4, which indicates that up to 5% of variations observed in the sampled insulin concentrations could be due the perifusion setup and assay. The differences in NaFl concentration between the different drops may be due to the small flow-rate differences between the peristaltic tubes, which were 15.0 μL min^–1^, 15.8 μL min^–1^, 15.8 μL min^–1^, and 15.9 μL min^–1^ for drops 1–4, respectively.

### Modeling of Insulin Release From Islets in Hanging Drops

**FIGURE 5:**
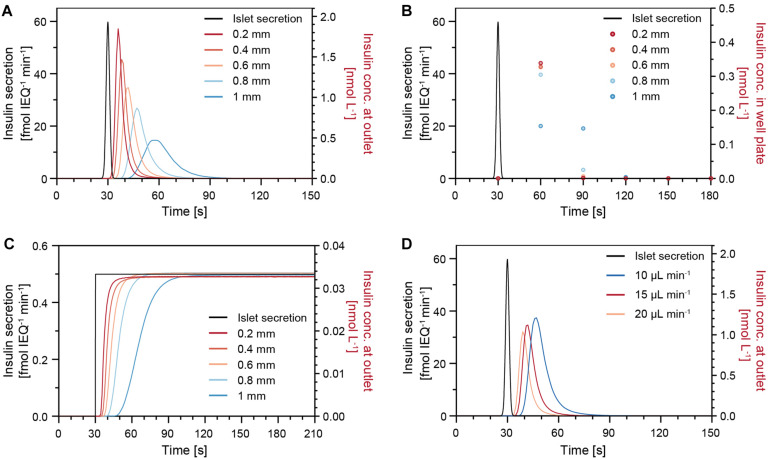
Modeling results of the insulin concentration profiles at the chip outlet after a sharp 1-s-long insulin secretion burst from an islet microtissue (black trace) for different parameters. **(A)** Different drop heights with an islet positioned in the center of the drop at a perifusion rate of 15 μL min^–1^. **(B)** Insulin concentration measured in the well plate for a sampling rate of 30 s. **(C)** Modeling results for constant and continuous insulin secretion at different drop heights with an islet positioned in the center of the drop at a perifusion rate of 15 μL min^–1^. **(D)** Different perifusion rates in a 0.6 mm drop with an islet positioned in the center of the drop.

The insulin release of single human islets exhibit characteristic features, such as a pronounced first phase and distinct oscillations in the second phase (Misun et al., 2020). In order to understand how well short secretion events of an islet can be captured, a sharp 1-s-long insulin secretion burst was simulated (Figure 5). The concentration of insulin at the chip outlet was calculated for different parameters including varying drop heights, perifusion rates, and islet positions. Figure 5A shows that, under a constant flow rate of 15 μL min^–1^, changes in drop height can have a significant effect on the transport of insulin. The analyte dispersion is more pronounced for increasing drop heights, which cause a delay of insulin to reach the chip outlet and a lower peak insulin concentration. For instance, in a small drop of 0.2 mm height, most of the secreted insulin reaches the outlet within 30 s after the secretion burst, but it takes about 60 s in a 1-mm-height drop. Figure 5B shows the expected insulin concentrations in the well plate if samples were collected at 30 s intervals and in phase with the islet secretion burst. For smaller drops of 0.2–0.6 mm height, the sampled concentration in the wells is similar, i.e., most of the secreted insulin is collected in the first sample, but the concentration can change significantly upon increasing drop size. Therefore, it is important to work with rather small drops to minimize dispersion and dilution of the secreted analyte, here insulin. However, for the assumption of continuous and constant insulin secretion, different drop heights would not affect the concentration at the outlet (Figure 5C).

Figure 5D illustrates how different perifusion rates can affect the insulin concentration at the outlet. The insulin, secreted by the islets, reaches the outlet faster at higher perifusion rates in comparison to lower perifusion rates. In addition, small deviations of the islet position, e.g., positions that are 200 μm off the drop center, will result in slight temporal shifts of the insulin concentration curves (Supplementary Figure 4). Considering the facts that the samples are collected at 30-s intervals and that the temporal shifts are not significant, small deviations in the islet position can be tolerated during a perifusion experiment. It is, however, of great importance to ensure a constant drop size and perifusion rate throughout an experiment and between experiments to achieve comparable measurements.

### Parallel FlowGSIS

**FIGURE 6:**
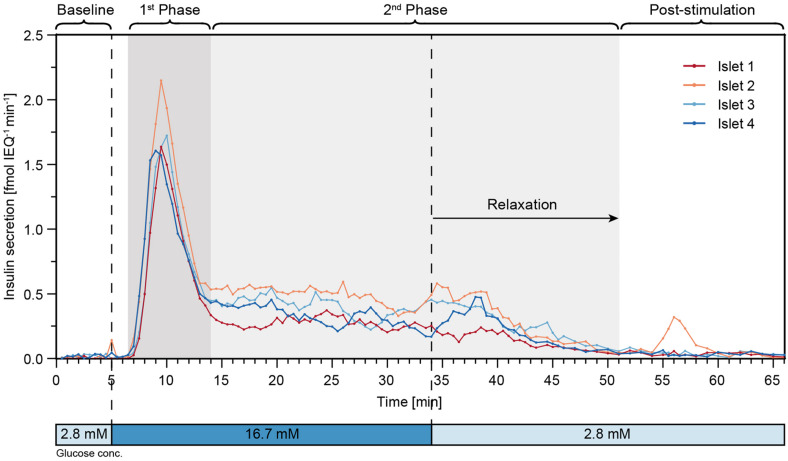
High-resolution microfluidic FlowGSIS measurements of four single islet microtissues in parallel. A change from low (2.8 × 10^–3^M) to high (16.7 × 10^–3^M) glucose concentrations stimulated similar biphasic insulin secretion responses in all four islets. Samples were continuously taken every 30 s between 0 and 45 min, and every 60 s between 45 and 66 min.

Finally, perifusion of islet microtissues was performed with the hanging-drop chip. Single islets from the same donor were loaded into each of the four islet drops. Figure 6 shows the GSIS response of the islet microtissues. The islets responded in a similar manner to the glucose stimulation. They featured a relatively low baseline secretion averaging to 0.02 ± 0.01 fmol IEQ^–1^ min^–1^. The islets in drop 2 and drop 4 showed a small peak secretion at 5 min. After switching to high glucose levels, it took about 1.5 min for the islets to get into the first phase, and the peak secretion occurred at 9–10 min. The peak secretion rates were 1.64 fmol IEQ^–1^ min^–1^, 2.15 fmol IEQ^–1^ min^–1^, 1.72 fmol IEQ^–1^ min^–1^, and 1.61 fmol IEQ^–1^ min^–1^ for islets 1–4 respectively. The second phase of insulin secretion began around 14 min. The average secretion levels in the second phase, between 14 min and 34 min, were 0.27 ± 0.04 fmol IEQ^–1^ min^–1^ for islet 1, 0.49 ± 0.07 fmol IEQ^–1^ min^–1^ for islet 2, 0.40 ± 0.08 fmol IEQ^–1^ min^–1^ for islet 3, and 0.32 ± 0.08 fmol IEQ^–1^ min^–1^ for islet 4. The switch to low glucose levels occurred at 34 min, and relaxation time for the islets to get back to baseline secretion levels was about 17 min. The post-stimulation phase started at 51 min, featuring a mean secretion of 0.03 ± 0.02 fmol IEQ^–1^ min^–1^ between 60–66 min for all four islets.

## Discussion and Conclusion

Islet perifusion systems allow for studying time-resolved insulin release of pancreatic islets in a GSIS scenario. Many islet perifusion technologies have been presented before, but they either have limitations in the readout or require complex instrumentation and experimental setups, which limit their usage outside of academia. To address these limitations, we developed a new generation of a microfluidic hanging-drop-based islet perifusion platform, based on a design that was used to resolve the details of the insulin secretion dynamics of a single pancreatic islet (Misun et al., 2020). We increased the experimental throughput fourfold and we simplified the system operation, as a microscope is no more needed. Furthermore, we used commercially available components, and miniaturized a standard ELISA assay that could be directly used with the samples without any further dilution steps. Downscaling of the ELISA reduced the assay costs, and the avoidance of dilution steps increased the quality of the assay, as the risk of pipetting errors was reduced.

We connected four islet drops symmetrically to a common inlet, which allowed for simultaneous stimulation of and sampling from four individual islets in parallel. A continuous and uninterrupted flow at a constant flow rate toward the islets was achieved and ensured stable flow conditions and a constant liquid volume in the chip over the duration of the assay.

We integrated a needle-type valve into a control drop to regulate and control the volume of all hanging drops in the fluidic network. The size of the four islet drops changed synchronously in response to the withdrawal of excess medium through the needle-type valve as a consequence of continuous pressure equilibration through the liquid phase. The withdrawal of medium through the needle valve whenever the drop height surmounted the needle length resulted in fluctuations in the sample drop sizes. However, we found that the deviations in drop size were relatively small, so that the drop heights remained approximately constant throughout the perifusion experiment. Since the fluctuation intervals ranged mostly between 6 and 9 s, and the sampled concentrations were integrated over 30 s, the fluctuations do not affect the analyte concentration that is released from the islets into the drop and sampled. We observed that the perifusion system introduced less than 5% variation in the measured concentrations.

Based on the numerical models, we observed that different drop heights, perifusion rates, and islet positions can affect dispersion and dilution of the released insulin. However, the effects of variation in perifusion rate and islet position on the sampled insulin concentrations is minimal, in particular considering 30 s sampling intervals. Nevertheless, it is advisable to keep constant drop height, perifusion rate, and islet position in all GSIS experiments to obtain reproducible and comparable experimental results. Moreover, we found that it is beneficial to have relatively small and well-defined drops. A small drop with a height of 0.6 mm or less ensures a quick sampling of the released analytes with minimal dilution and low dispersion; a well-defined drop shape ensures that the islets remain at a stable position at the bottom of the drop.

We presented a stable and robust perifusion system that could perform simultaneous GSIS assays on four individual islet microtissues at a sampling resolution of 30 s. Islets coming from the same donor showed very similar GSIS responses. A baseline secretion was obtained after 30 min of equilibration at low glucose levels. Fast changes in insulin release were observed in response to stimulation with high glucose levels, and all islets secreted insulin in a biphasic manner with clear separation between the first and second phase of insulin release. A delayed response after switching back to low glucose levels was observed. The measured insulin concentrations showed low sample-to-sample variation, which is due to a precise liquid handling with stable drop volumes, equal flow rates, and defined sampling volumes from all four drops hosting the islets. The reproducibility of the FlowGSIS results and dynamics across several technical replicates in the same run shows that the presented hanging-drop-based microfluidic platform could be a useful tool for studies of human islets. Yet, a clear definition of the different phases still needs to be established for the analysis and extraction of data in a reliable and reproducible manner. For instance, the start and end of the first phase and the duration of the second phase can be determined either according to the time points of the stimulations or according to the time points when changes in the insulin release occur.

The presented microfluidic chip features a modular design including T-junctions and symmetrically designed channels so that the experimental throughput may be further increased to, e.g., eight islet drops. However, the increase in hydraulic channel resistance and perifusion rates may be limiting factors. For instance, higher channel resistances may prolong the time required for pressure equilibration between the hanging drops, which may result in a collapse of the fluidic system so that, e.g., one of the drops becomes too big and drips off.

The hanging-drop perifusion system can be used to measure other hormones, released by islet microtissues or other 3D cell models. For instance, islets also release glucagon and somatostatin in small quantities in response to glucose stimulation (Braun et al., 2009; In’t Veld and Marichal, 2010). However, currently there is no suitable ELISA available that is sensitive enough to quantify small concentrations of these hormones in small sample volumes.

In conclusion, the hanging-drop-based islet perifusion system enables parallel GSIS measurements and automated sampling from individual islets. With increased experimental throughput and a robust operating system, this newly devised platform offers the potential to study insulin-release dynamics of single human pancreatic islets or rodent pancreatic islets for use in drug screening and basic research.

## Data Availability Statement

The raw data supporting the conclusions of this article will be made available by the authors, without undue reservation.

## Author Contributions

PW and PM contributed to conception and design of the study. PW performed the experimental research and data acquisition. PW and PM interpreted the data. PW and NR worked on the numerical models. PW, PM, and AH contributed to writing and editing of the manuscript. All authors contributed to the article and approved the submitted version.

## Conflict of Interest

The authors declare that the research was conducted in the absence of any commercial or financial relationships that could be construed as a potential conflict of interest.
