# Supplementary material for: A Microfluidic Hanging-Drop-Based Islet Perifusion System for Studying Glucose-Stimulated Insulin Secretion From Multiple Individual Pancreatic Islets
Source: Front Bioeng Biotechnol. 2021 May 12;9:674431. doi: 10.3389/fbioe.2021.674431 (PMC8149801; doi:10.3389/fbioe.2021.674431)
Supplement: Supplementary file 2 [file Image_1.pdf]

SUPPLEMENTARY MATERIAL

SUPPLEMENTARY FIGURES

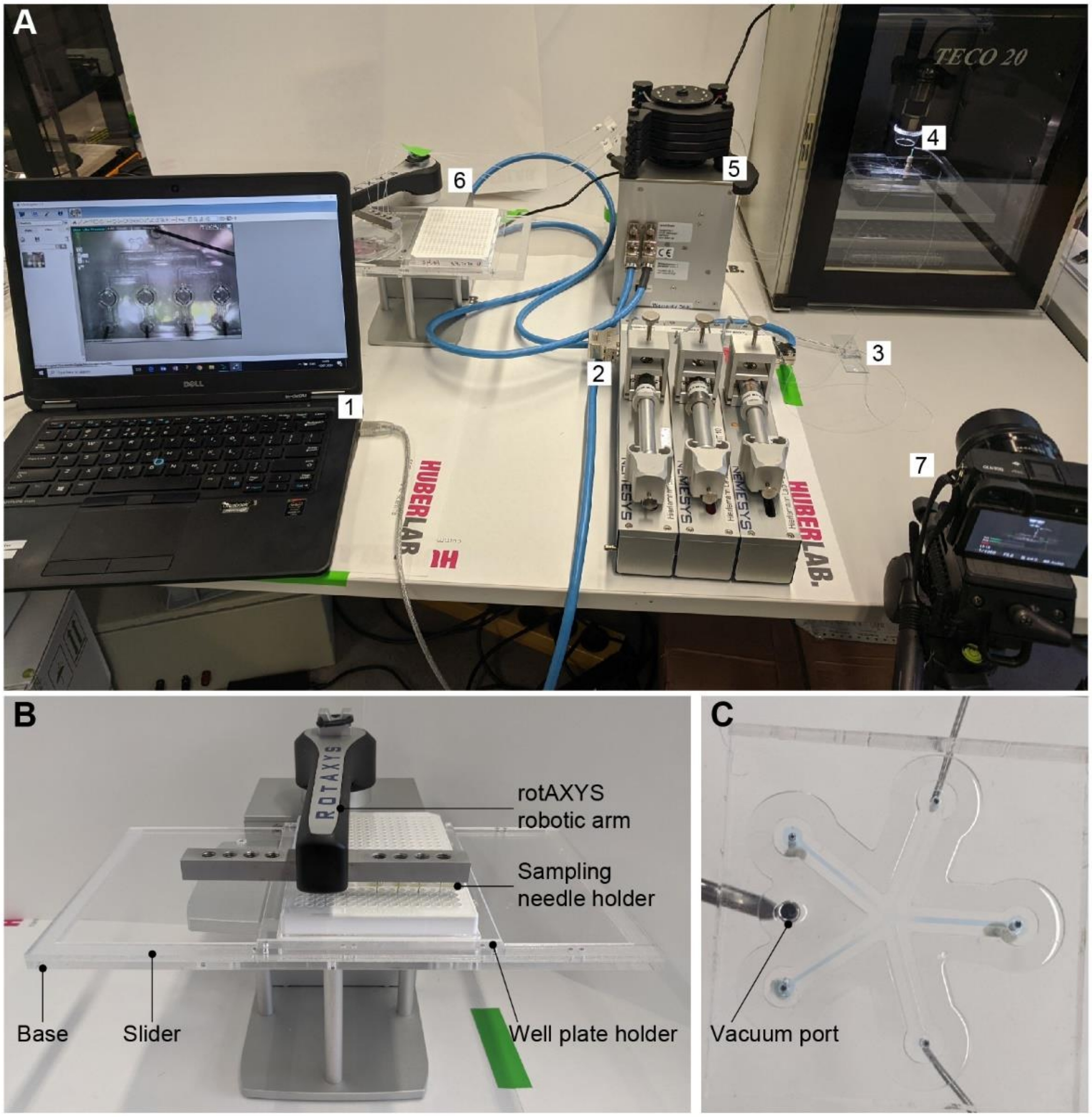

FIGURE S1 | Images of the experimental setup. **(A)** FlowGSIS setup: (1) laptop running Qmix Elements and recording the videos from the Dino-Lite camera, (2) neMESYS syringe pumps loaded with 10 mL glass syringes, (3) microfluidic splitter connected to vacuum, (4) incubator with microfluidic chip placed under the Dino-Lite camera, (5) peRYSIS peristaltic pump, (6) rotAXYS robotic arm with sampling well plate, and (7) camera taking side-view videos of the chip. **(B)** rotAXYS setup up with customized parts for automated sampling. **(C)** Microfluidic splitter with five fluidic ports and one vacuum port. The vacuum applied around the fluidic channels helps to prevent bubble formation in the media reservoirs and channels, thus ensuring bubble-free chip perifusion.

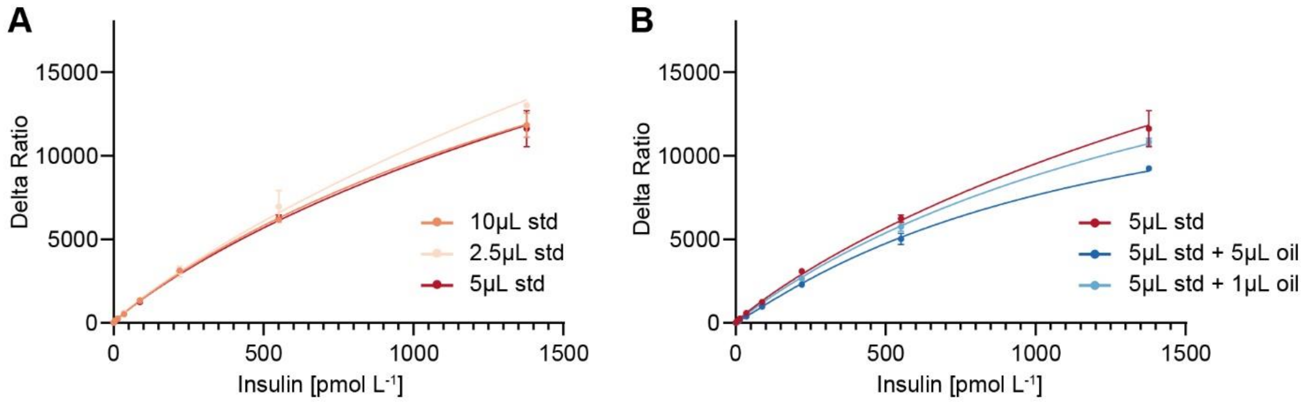

FIGURE S2 | Characterization of the Insulin ELISA assay. Different volumes of insulin standards and assay reagents were mixed at a ratio of 1:1, with 10 µL of sample being the standard recommended volume of the assay. (**A**) Miniaturization of the ELISA assay, showing the influence of the sample volume. (**B**) Comparison of ELISA assay of samples, which were not covered with oil and assays of samples, which were covered with 1 or 5 µl oil, showing the influence of the oil phase on the readout.

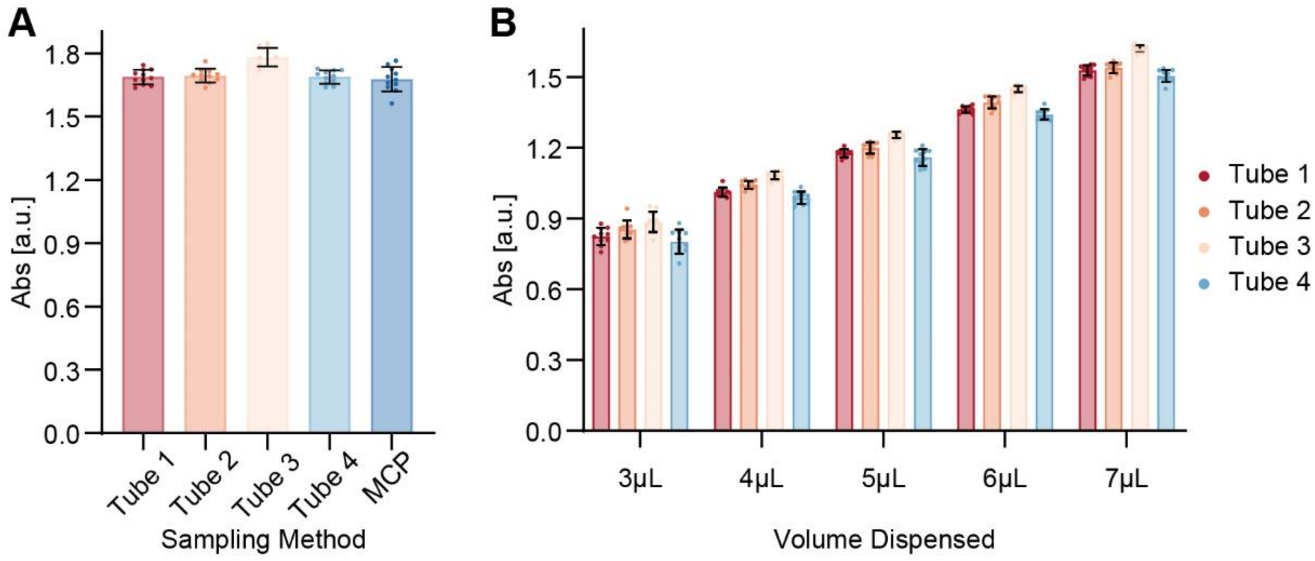

FIGURE S3 | Characterizations of liquid sampling. **(A)** Accurate sampling with peRYSIS peristaltic pump and rotAXYS robotic arm. 5 µL of amaranth solution were loaded into the wells using the rotAXYS and peRYSIS with four peristaltic tubes and with a multichannel pipette (MCP) (n=11). The wells were preloaded with 5 µL mineral oil. **(B)** Precise sampling with peRYSIS peristaltic pump and rotAXYS robotic arm into a 384 well plate. Different volumes of amaranth were loaded into the wells with the peRYSIS pumping at 15 µL/min by varying the dispensing time in each well with the rotAXYS robotic arm (n=11). Absorbances of the samples were measured at 520 nm wavelength.

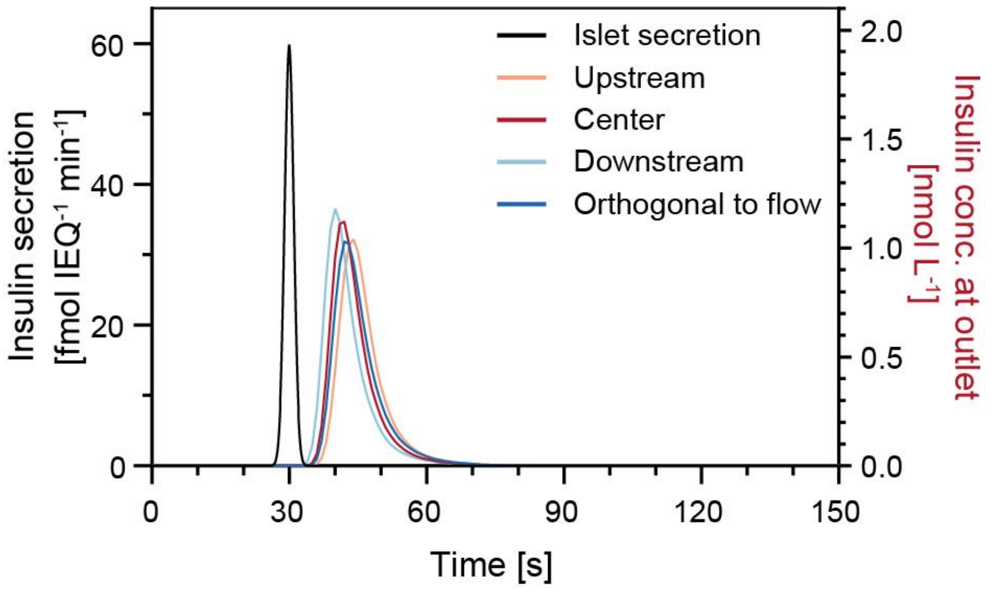

FIGURE S4 | Modeling results of the insulin concentration profiles at the chip outlet after a sharp 1-s-long insulin secretion burst from an islet microtissue (black trace) for different islet positions in a 0.6-mm drop for a perifusion rate of 15 μL min^{-1}. The islets were displaced by 200 μm from the center either upstream, downstream, or orthogonal to the flow.
